# Differential abscopal effect in extracranial and intracranial lesions after radiotherapy alone for vertebral bone metastasis of unknown primary: a case report

**DOI:** 10.1186/s13256-022-03321-x

**Published:** 2022-03-06

**Authors:** Yojiro Ishikawa, Rei Umezawa, Takaya Yamamoto, Noriyoshi Takahashi, Kazuya Takeda, Yu Suzuki, Keiichi Jingu

**Affiliations:** 1 Department of Radiation Oncology, Tohoku University Graduate School of Medicine, 1-1 Seiryo-chou, Aoba-ku, Sendai, MIyagi 980-8574, Japan; 2 Division of Radiology, Faculty of Medicine, Tohoku Medical and Pharmaceutical University, 1-15-1 Fukumuro, Miyagino-ku, Sendai, 983-8536 Japan

**Keywords:** Abscopal effect, Unknown primary, Palliative radiotherapy

## Abstract

**Background:**

The abscopal effect is a phenomenon in which a tumor located far from irradiated lesions regresses. We have experienced a case in which both intracranial and extracranial lesions showed an abscopal effect after radiotherapy for spinal metastases of unknown primary. We report the differential abscopal effect in extracranial and intracranial lesions.

**Case presentation:**

A 57-year-old Japanese man was diagnosed with multiple lung nodules, bone metastases, and brain metastases. The results of pathological examination at the previous hospital he visited suggested adenocarcinoma of the lung. However, there was a possibility that the biopsy specimen was inadequate. Radiation therapy was performed on the ninth thoracic vertebra for a total dose of 39 Gy in 13 fractions because the lesion in the ninth thoracic vertebra was destructively extending. After thorough examination, the primary lesion could not be identified, and we made diagnosis of cancer of unknown primary. The patient did not want to receive systemic chemotherapy; however, all of the lesions except for the brain metastases had spontaneously shrunk 2 months after radiation therapy. Although the brain metastases had partially shrunk, whole-brain radiotherapy for a total dose of 36 Gy in 12 fractions was performed. Fifteen months after initial radiation therapy, the brain metastasis recurred, and Gamma Knife radiosurgery was additionally performed. The brain metastases disappeared after the radiosurgery. During a period of 30 months after radiation therapy for the ninth vertebra, the lesions of the trunk all maintained their shrinkage without systemic chemotherapy. Right cervical lymph node metastasis and brain metastases occurred 30 months after the initial radiation therapy. A biopsy of the right cervical lymph node led to the diagnosis of clear cell carcinoma. Although we considered additional radiation therapy or chemotherapy, the patient died 3 months after the progression of recurrence lesions.

**Discussion and conclusions:**

We report a rare case in which radiotherapy alone for an extracranial metastatic lesion of a vertebra resulted in an abscopal effect on both extracranial and intracranial lesions. Notably, the abscopal effect in the intracranial lesions was weaker than that in the extracranial lesions.

## Background

The abscopal effect is a phenomenon in which a tumor located far from irradiated lesions regresses. In general, intracranial lesions are not susceptible to the abscopal effect due to the presence of the blood–brain barrier (BBB), and there have been no reports of an abscopal effect of radiotherapy of extracranial lesions on intracranial lesions.

The spine is a common site of metastasis of malignant tumors; however, there have been few reports of an abscopal effect, and only two cases of treatment with radiotherapy alone have been reported [1, 2]. In this report, we describe a case in which both intracranial and extracranial lesions showed an abscopal effect after radiotherapy of spinal metastases, and we report the differences in the clinical courses of intracranial and extracranial lesions.

## Case presentation

**Fig. 1:**
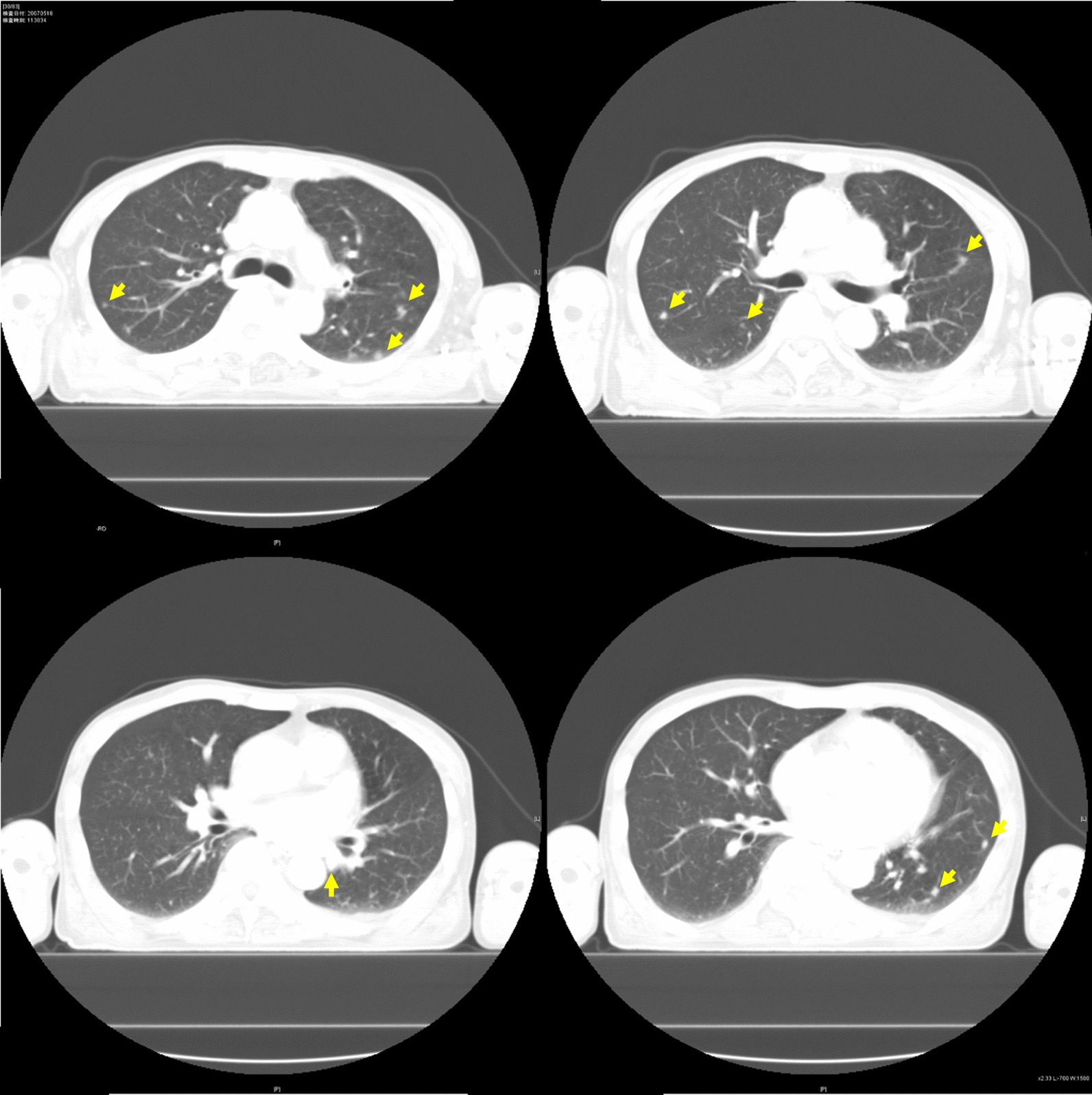
Computed tomography showed multiple nodules in bilateral lungs (yellow arrows)

**Fig. 2:**
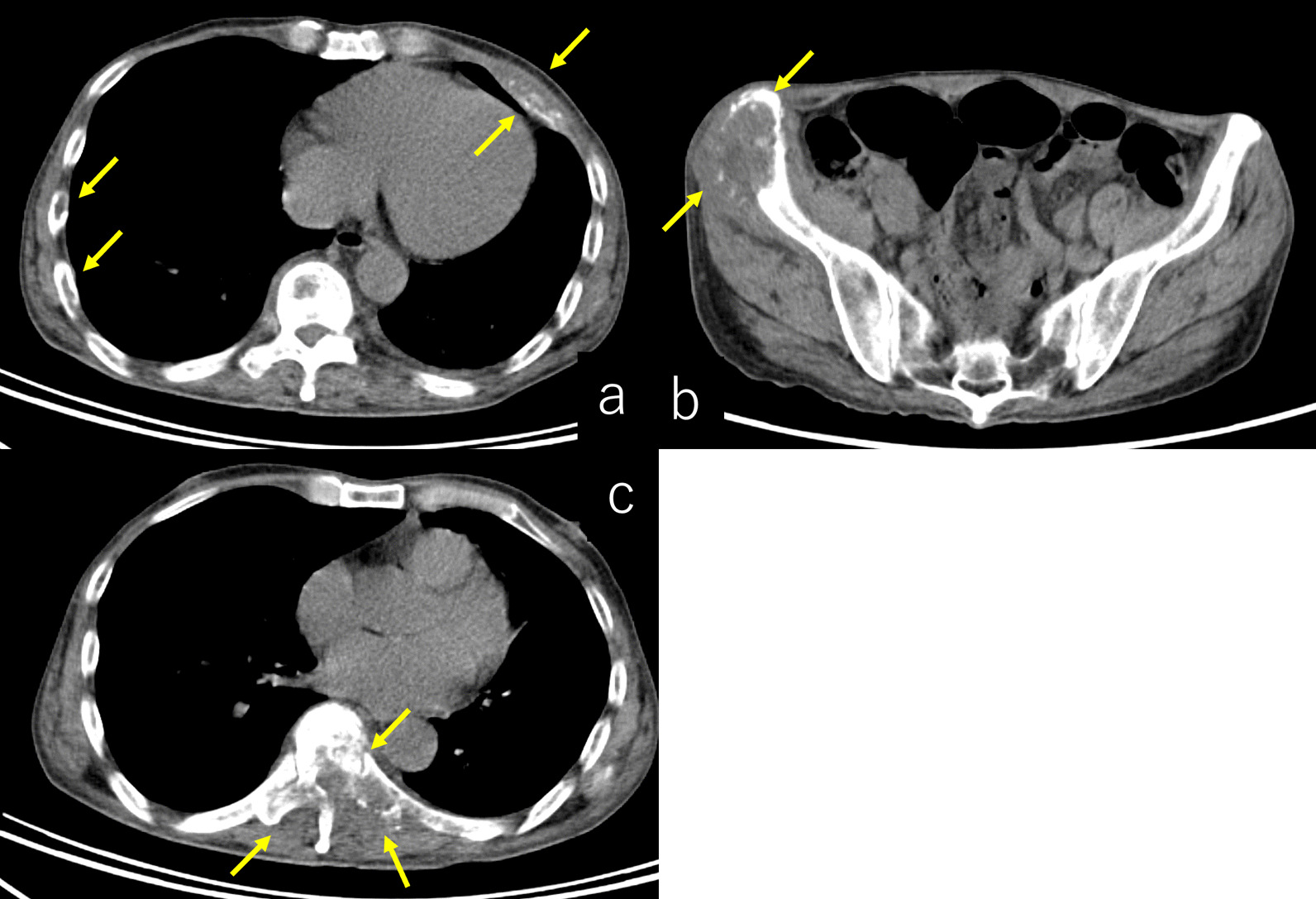
Computed tomography showed lesions in left eighth rib (**a**), right iliac bone (**b**), and ninth thoracic vertebra (**c**) (yellow arrows)

A 57-year-old Japanese man complained of left chest pain 2 months before visiting the previous hospital. He had lost 8 kg of weight in 2 months. He had no previous medical history and was not taking any medications. He had smoked 20 cigarettes a day for 40 years (Brickman index 800). It was revealed that he had a family history of lung cancer in his father. In the previous hospital, computed tomography (CT) showed multiple nodules in bilateral lungs (Fig. 1) and lesions in the left eighth rib, right iliac bone, and ninth thoracic vertebra (Fig. 2). The results of a biopsy of the metastatic rib lesion at the previous hospital indicated a diagnosis of malignancy (suspected adenocarcinoma of the lung). Additional pathological diagnostic tests were considered, but they were not done because the biopsy sample was inadequate. The imaging findings of multiple nodules in the lungs and multiple metastases in the bones and the pathological diagnosis led to the suspicion of lung cancer at the previous hospital. Prostate cancer and renal cancer were suspected due to the frequent bone metastases. It was also determined that he needed further examination for cancer of the gastrointestinal tract, liver, pancreas, and gall bladder.

**Fig. 3:**
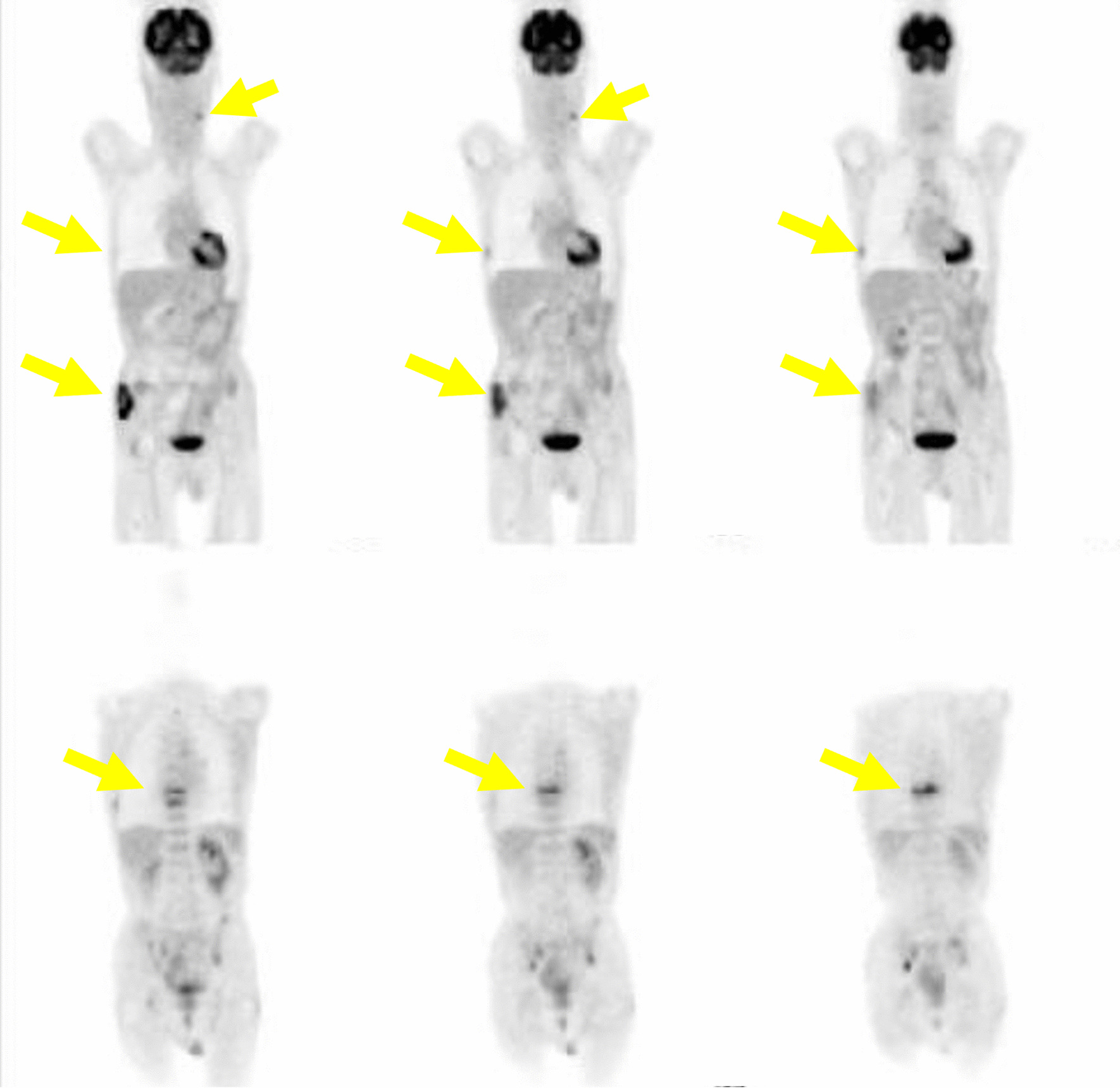
Positron emission tomography–computed tomography (PET-CT) performed in our hospital showed uptake in the left eighth rib, ninth vertebra, right iliac bone, and left neck lymph node with maximum standardized uptake values (SUVmax) of 2.8, 6.0, 4.1, and 3.5 (yellow arrows), respectively

Positron emission tomography–computed tomography (PET-CT) performed in our hospital showed ^18^F-2-fluoro-2-deoxy-d-glucose (FDG) uptake in the left eighth rib, ninth vertebra, right iliac bone, and left neck lymph node with maximum standardized uptake values (SUVmax) of 2.8, 6.0, 4.1, and 3.5, respectively. The pulmonary nodules did not show high uptake (Fig. 3).

**Fig. 4:**
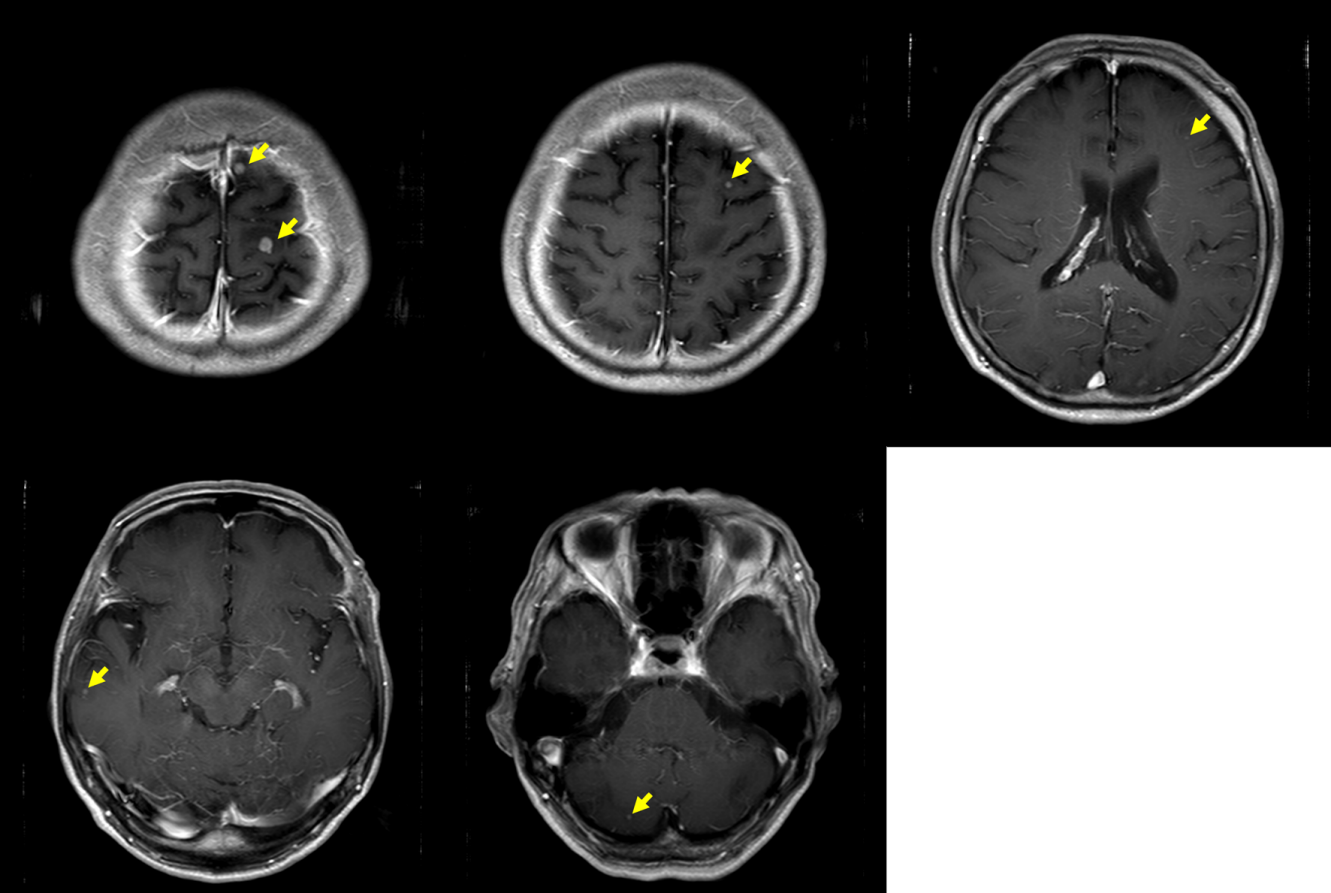
Magnetic resonance imaging showed multiple brain metastases (yellow arrows) but no significant neurological symptoms

Magnetic resonance imaging (MRI) showed multiple brain metastases but no significant neurological symptoms (Fig. 4). There were also no apparent masses in the liver, pancreas, or gall bladder. Sputum cytology showed no obvious malignancy, and biopsy could not be performed because of the small sizes of the pulmonary lesions. Upper gastrointestinal endoscopy showed an elevated lesion in the duodenum, and a biopsy was performed, but there were no obvious malignant findings due to suspected hyperplasia of Brunner’s gland. Ultrasonography of the kidneys and bladder was performed because of gross hematuria; however, no obvious tumor was found. Urine cytology was negative. Results of laboratory investigations of tumor markers are presented in Table 1. The levels of CA15-3 and KL-6 were elevated. Breast cancer was suspected on the basis of the level of CA15-3, but imaging findings were negative. Despite a thorough examination, the primary tumor was not confirmed. The patient was treated as a patient with cancer of unknown primary.

**Table 1:** Tumor marker values

Tumor marker	Value	Normal range
SCC	0.9 ng/ml	0–1.5 ng/ml
CEA	5.9 ng/ml	0–5 ng/ml
ProGRP	20.5 ng/ml	0–46 ng/ml
NSE	17.4 ng/ml	0–10 ng/ml
CA19-9	15 U/ml	0–37 U/ml
CA125	20.2 U/ml	0–39 U/ml
CA15-3	286 U/ml	0–28 U/ml
AFP	4.0 ng/ml	0–20 ng/ml
HCG	1.0 mIU/ml	0–3 mIU/ml
PSA	0.682 ng/ml	0–4.0 ng/ml
KL-6	2330 U/ml	105–435 U/ml

*SCC* squamous cell carcinoma antigen, *CEA* carcinoembryonic antigen, *Pro-GRP* pro-gastrin releasing peptide, *NSE* neuron-specific enolase, *CA* carbohydrate antigen, *AFP* alpha-fetoprotein, *HCG* human chorionic gonadotropin, *PSA* prostate-specific antigen, *KL-6* Krebs von den Lungen-6

**Fig. 5:**
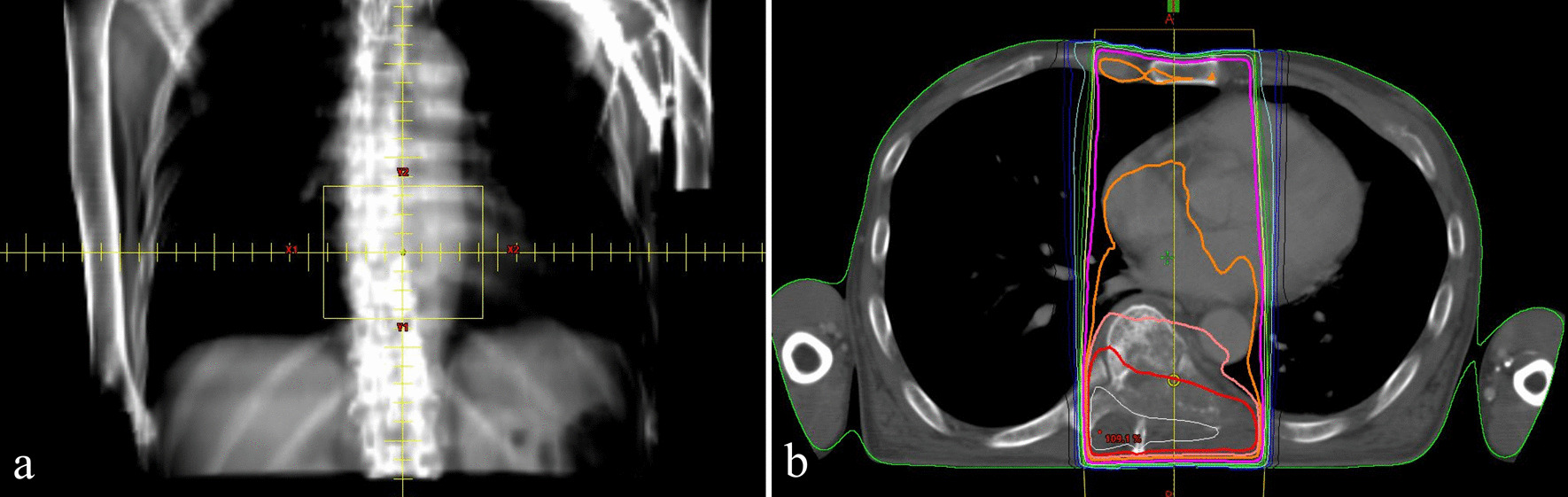
Irradiation field (**a**) and dose distribution (**b**) of radiation therapy for the ninth thoracic vertebra for a total dose of 39 Gy in 13 fractions

**Fig. 6:**
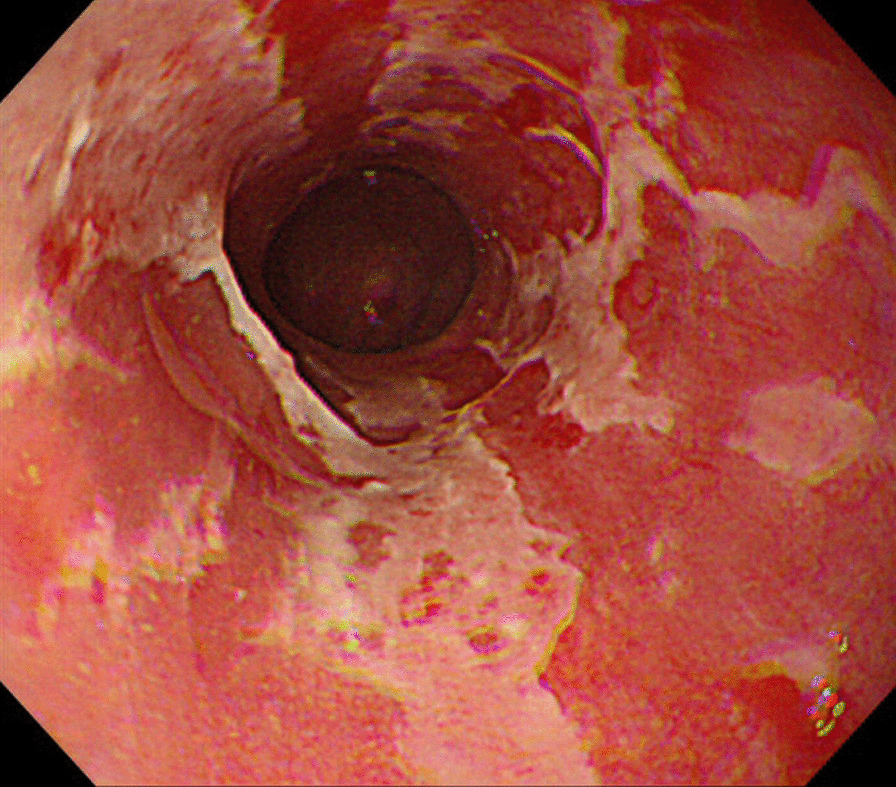
Radiation esophagitis of grade 2 was observed 2 weeks after radiotherapy

The lesion in the ninth thoracic vertebra was destructively extending to the ninth vertebral body. Although a diagnosis had not been made, it was determined that treatment was necessary as soon as possible. On the other hand, the brain metastasis was asymptomatic; we did not immediately perform whole-brain radiotherapy (WBRT). Therefore, an explanation was given to the patient and we obtained consent to perform palliative irradiation to the ninth thoracic vertebra. Although the patient had multiple metastases, he had a relatively good Eastern Cooperative Oncology Group performance score of 1, and considering the fact that the patient was untreated, irradiation was planned within the range not exceeding the tolerance dose of the spinal cord. Palliative radiation therapy (RT) was performed on the ninth thoracic vertebra for a total dose of 39 Gy in 13 fractions. The irradiation field is shown in Fig. 5. The treatment was completed without any serious side effects requiring suspension or discontinuation of irradiation; however, radiation esophagitis was observed 2 weeks after RT (Fig. 6).

**Fig. 7:**
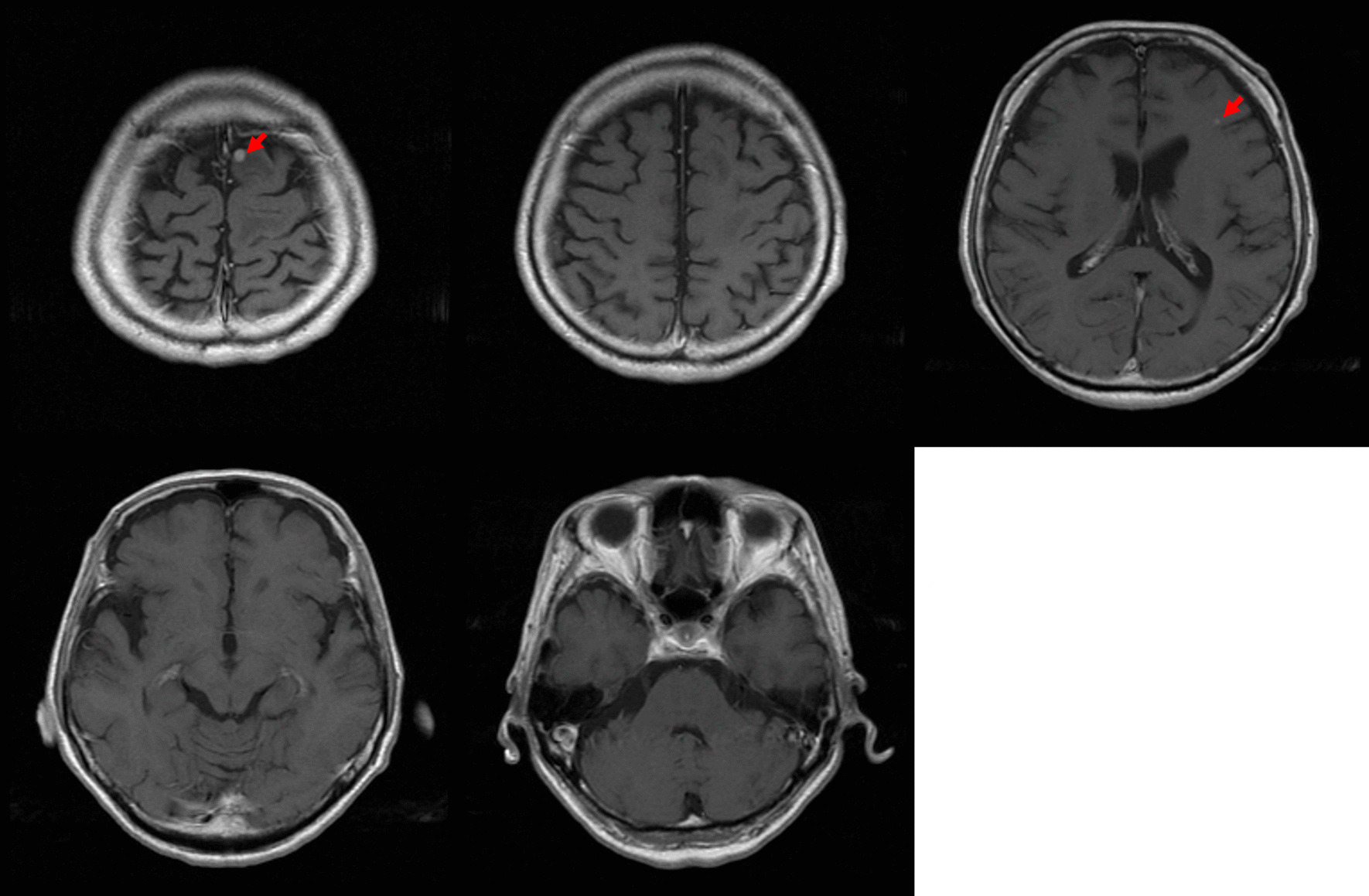
Magnetic resonance imaging for the purpose of whole-brain radiotherapy showed that almost all of the tumors had spontaneously shrunk 1 month after radiotherapy for the ninth vertebra. The two tumors in the frontal lobe had not shrunk or grown (red arrows)

**Fig. 8:**
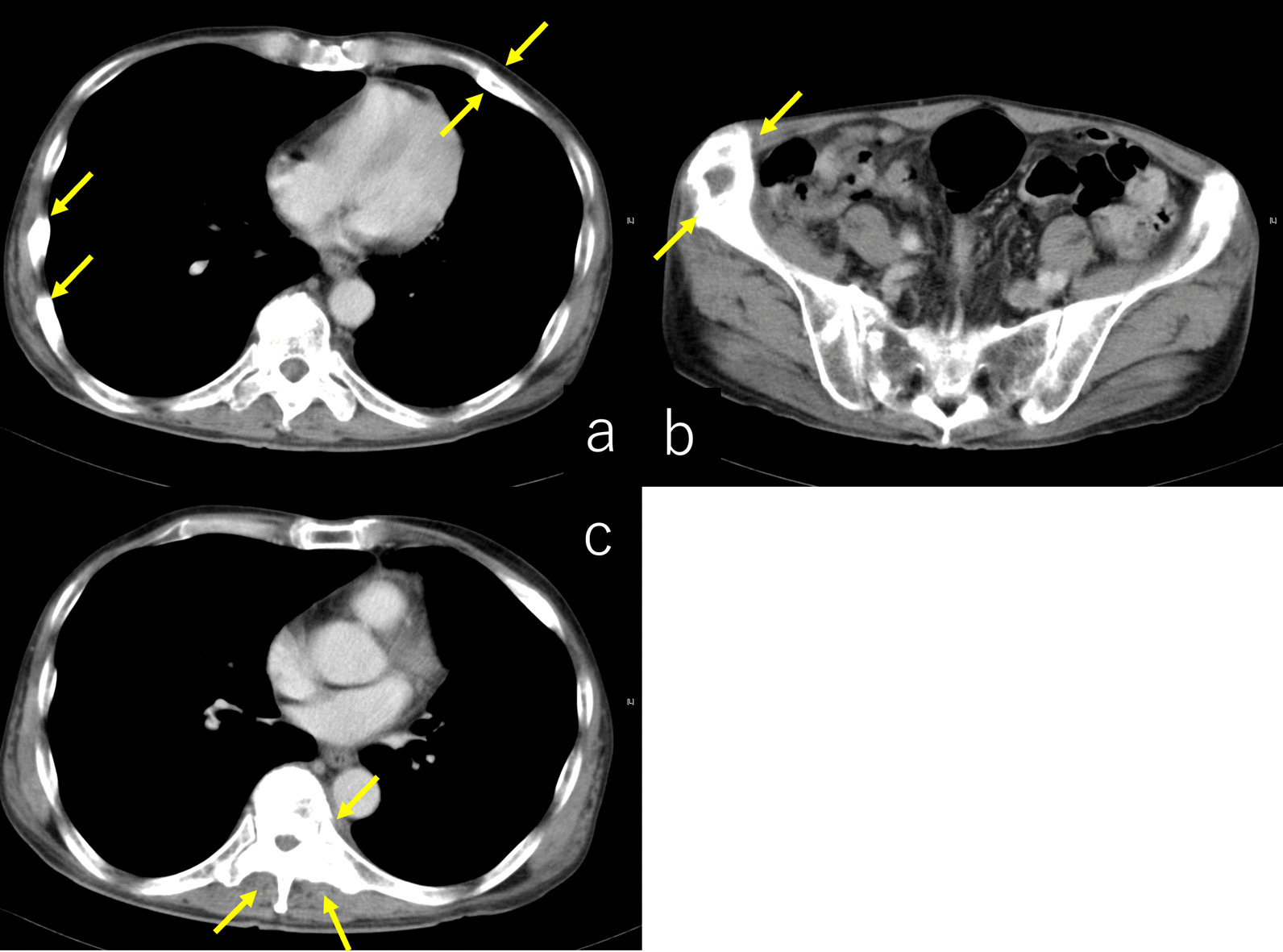
Computed tomography showed shrinkage of the lesion in the left eighth rib (**a**), right iliac bone (**b**), and ninth thoracic vertebra (**c**) (yellow arrows)

**Fig. 9:**
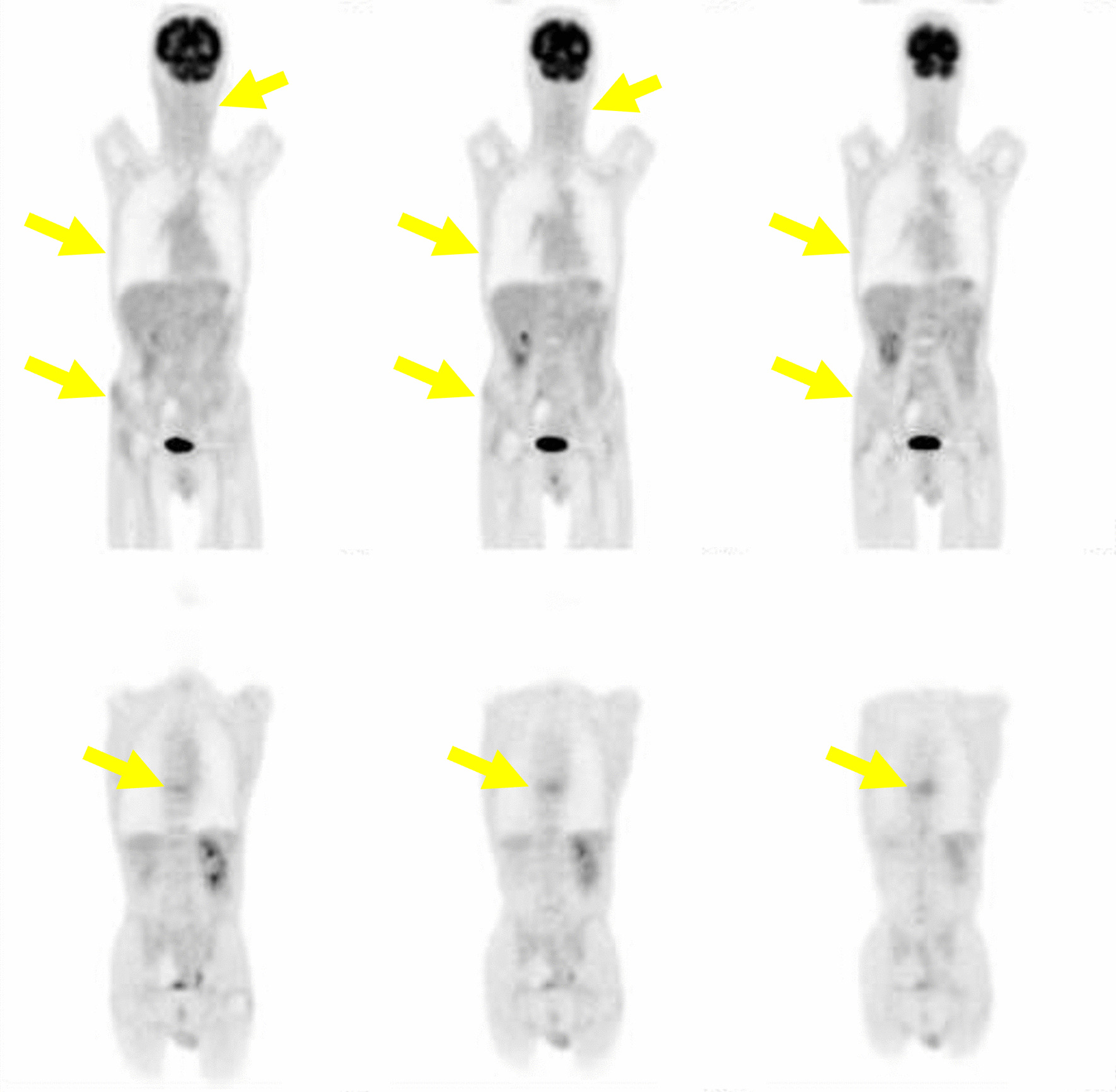
Positron emission tomography–computed tomography showed shrinkage of all lesions except brain metastases and disappearance of uptake 1 month after radiotherapy for the ninth vertebra (yellow arrows)

Follow-up MRI examination for WBRT showed that almost all of the tumors had spontaneously shrunk 1 month after RT for the ninth vertebra (Fig. 7). Although the brain metastases had not disappeared, we decided to observe the patient and did not perform WBRT. CT and PET-CT showed shrinkage of all lesions except the brain metastases, and FDG uptake disappeared (Figs. 8 and 9). Examination of tumor markers revealed a CA15-3 level of 34 U/ml and KL-6 level of 254 U/ml. Because the lesions had shrunk, the primary lesion could not be determined. It was difficult to decide what type of chemotherapy was appropriate. In addition, the patient did not wish to receive additional treatment because the tumor size was maintained without treatment.

**Fig. 10:**
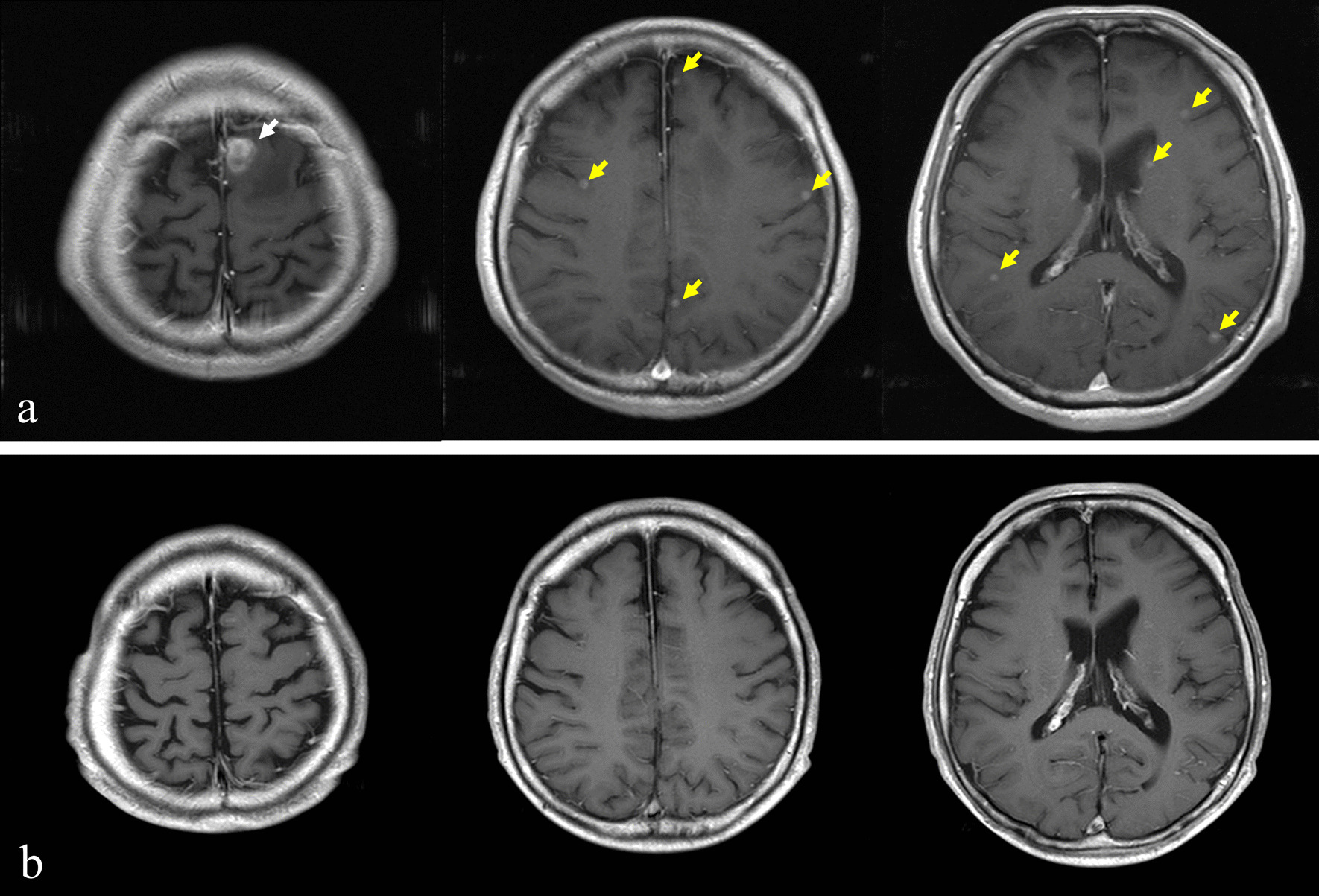
Magnetic resonance imaging showed multiple brain metastatic masses 3 months after radiotherapy for the ninth vertebra (**a**). The tumor in the frontal lobe was enlarged (white arrow), and numerous other metastases appeared (yellow arrows). Whole-brain radiotherapy (WBRT) was performed 2 months after the initial radiation therapy. After WBRT, all brain metastases disappeared (**b**)

Thereafter, the extracranial lesions continued to shrink without treatment; however, an MRI scan showed multiple brain metastases 3 months after RT for the ninth vertebra (Fig. 10a). WBRT for a total dose of 36 Gy in 12 fractions was performed 3 months after the initial RT. One month after WBRT, all of the brain metastases had disappeared (Fig. 10b). The patient did not want to have chemotherapy.

**Fig. 11:**
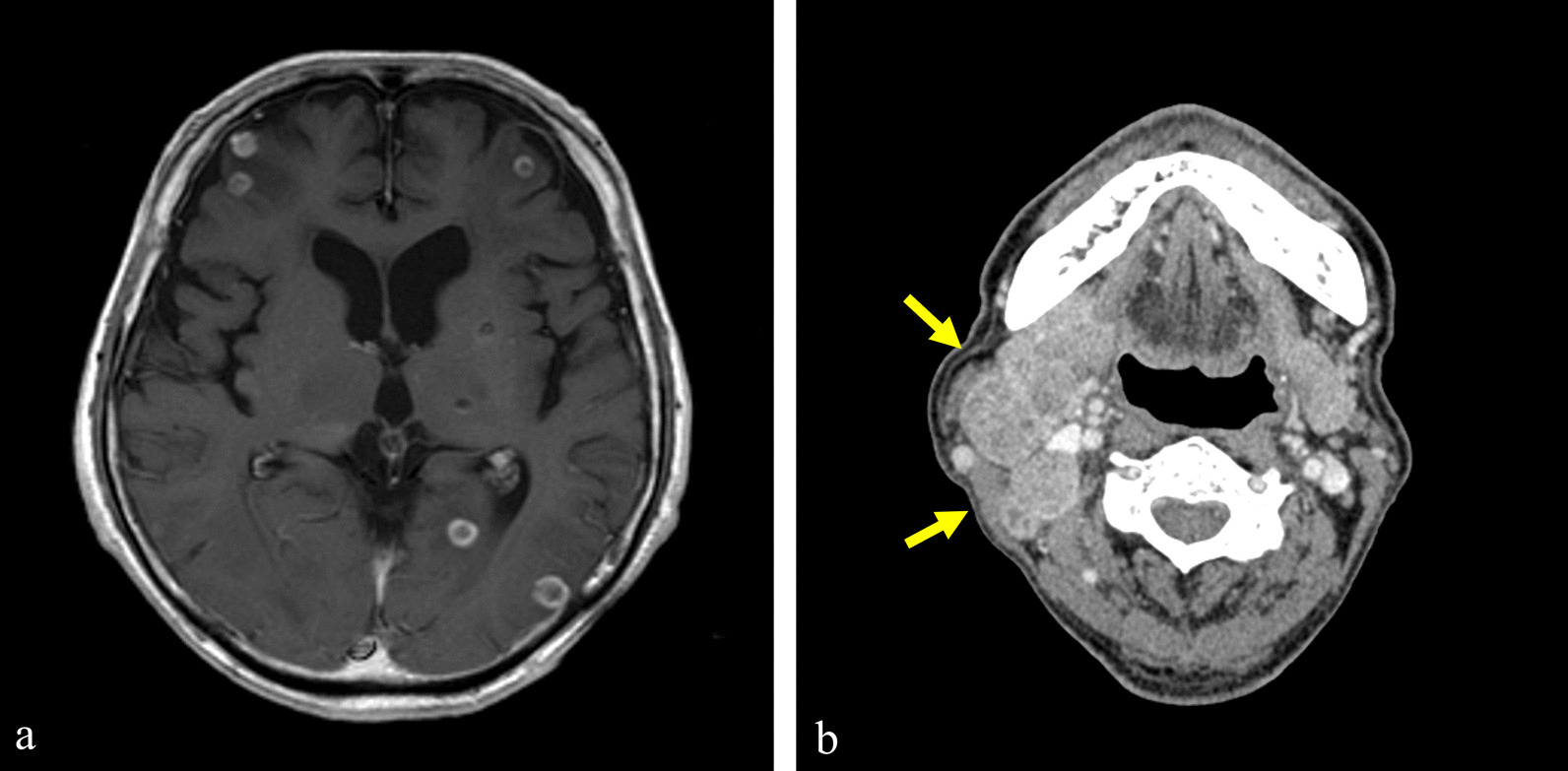
Fifteen months after whole-brain radiotherapy, brain magnetic resonance imaging showed multiple brain metastases (**a**) and computed tomography showed right cervical lymph node metastases (yellow arrows) (**b**)

Fifteen months after the initial RT (12 months after WBRT), MRI showed recurrence of brain metastasis (Fig. 11a). There were no lesions other than multiple brain metastases, and all of the lesions in the brain were less than 2 cm in size. We judged them to be an indication for Gamma Knife radiosurgery. Gamma Knife radiosurgery was therefore performed for 14 brain metastatic lesions, and MRI showed the disappearance of the lesions after Gamma Knife radiosurgery.

During a period of 30 months after RT for the ninth vertebra, the lung metastases maintained their shrinkage and the lesions in the iliac bone, ribs, and ninth vertebra and other lesions of the trunk all maintained their shrinkage. Thereafter, the right cervical lymph node metastasis showed rapid enlargement (Fig. 11b). The brain metastases also recurred again.

A needle biopsy of the right cervical lymph node metastasis led to the diagnosis of clear cell carcinoma (CCC). Immunostaining showed a positive pattern for AE1/AE3, vimentin, and EMA and a negative pattern for K7 and K20. In addition, CD10, CD15, PSA, TTF-1, S-100, CEA, HMB45, p63, CK14, and 34βE12 were all negative.

**Fig. 12:**
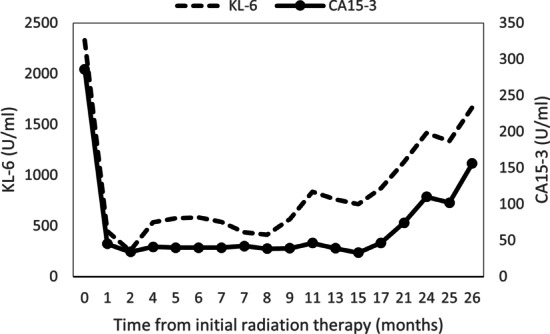
Changes in tumor marker values from the initial treatment

**Fig. 13:**
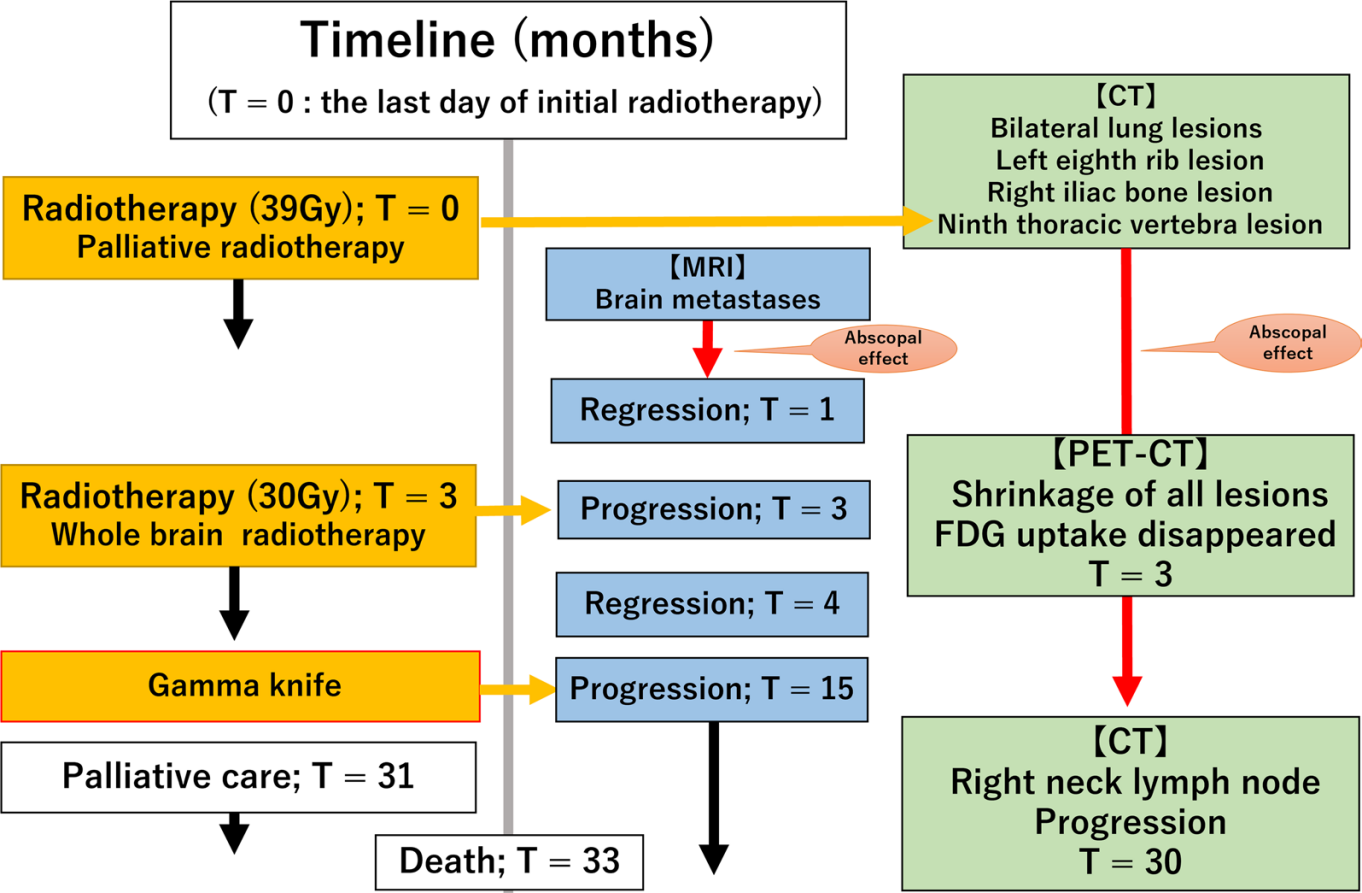
Timeline of the present case

There were marked elevations in levels of the tumor markers CA15-3 (156.6 U/ml, normal range: 0–28 U/ml) and KL-6 (1668, normal range: 105–435 U/ml) as was observed at the time of the initial treatment. The changes in tumor images and tumor marker values from the initial treatment are shown in Fig. 12. After the pathological diagnosis, radiotherapy to the right cervical lymph node metastasis and additional systemic chemotherapy were considered; however, the patient developed left lower body paralysis and disorientation due to the progression of brain metastasis. Three months after the progression of brain metastases, the patient died. An autopsy was not carried out. The timeline of the present case is shown in Fig. 13

## Discussion

In 1953, RH Mole first reported the abscopal effect as a phenomenon of spontaneous regression of tumors at a distance from the irradiated tissue [3]. In a review of a total of 46 cases of the abscopal effect from 1969 to 2014, it was shown that the median radiation dose was 31 Gy, the median follow-up period was 17.5 months, and the median time from radiation treatment to the onset of the abscopal effect was 2 months [4]. There has been an increasing number of studies on the abscopal effect since it was introduced in a paper by Postow *et al.* published in the *New England Journal of Medicine* in 2012 [5]. Recently, there has been an increasing number of reports on the combined use of immune checkpoint blockade therapy and RT [6].

The brain has its own immune system with a BBB that prevents lymphocytes from entering the brain [7]. It is difficult for immunogenic signaling molecules to travel across the BBB; however, a recent study in mice showed that there was a functional lymphoid system along the dural sinus [8]. Since it was reported that central nervous system-derived antigens induce immune responses in cervical lymph nodes, this immune privilege perspective has been under review [9].

It is possible that localized central nervous system radiation can disrupt the structural integrity of the BBB and increase its endothelial permeability, allowing free passage of immunogenic responses between the intracranial and extracranial compartments. In general, intracranial lesions are not susceptible to the abscopal effect from extracranial lesions due to the presence of the brain–blood barrier. In the brain, microglia function as immune cells and are capable of antigen presentation and phagocytosis [10]. When the BBB is disturbed by tumor formation, lymphocytes in circulating blood invade the brain from the area of tumor formation [6], but the relationship with the abscopal effect remains unclear.

To the best of our knowledge, there have been no reports, including clinical case reports, on brain tumor shrinkage or disappearance after RT alone for extracranial tumors. Ishiyama *et al*. reported an abscopal effect of 40 Gy in five fractions of stereotactic body radiotherapy for bone metastases and vertebral bone lesions after nephrectomy and stereotactic radiosurgery for brain metastases for renal cell carcinoma with diffuse metastases (bone, brain, lung, and mediastinum), which resulted in regression of lesions excluding brain metastases [1]. In the report by Ishiyama *et al.*, they hypothesized that the abscopal effect might not reach brain metastasis.

In our case, irradiation of an extracranial lesion caused an intracranial abscopal effect, but the response was not uniform throughout the brain. Some tumors did not show any abscopal effect. Furthermore, although the abscopal effect of the extracranial lesions was persistent, the intracerebral lesions relapsed within a few months. The irradiation on extracranial lesions may indicate that the abscopal effect on intracranial lesions is unlikely to occur or is weaker. Piercey *et al.* reported that a patient with metastatic melanoma, which progressed rapidly despite concomitant immune checkpoint blockade, showed both extracranial and intracranial abscopal effects after initiating palliative radiation to the axilla [11]. This suggests that some kinds of combination treatment may be necessary to induce an intracranial abscopal effect from extracranial lesions. To improve the prognosis of metastatic brain tumors in the future, it is important to clarify how tumor antigen-specific cytotoxic T lymphocytes and microglia in the circulating blood respond to tumors in the brain. Regarding the reason for the different effects between intracranial lesions, we speculate that some substance or factors that cannot break through the BBB may be involved in the abscopal effect. On the other hand, the immunosuppressive brain environment is not the sole hypothesis for the differential abscopal effect in the brain; the clonogenic divergence specific to brain metastasis and variations in the expression of antigens may also be involved. Brastianos *et al.* reported that divergent evolution was observed in all clonally related cancer samples for 86 matched brain metastases, primary sites, and normal tissues, with metastatic and primary sites having a common origin but continuing to evolve independently. In 53% of the cases, clinically useful changes were found in the brain metastases but were not detected in the matched primary site samples [12].

The present case is the second case of CCC in which an abscopal effect was observed after irradiation of the spine, and this is the first report of a pure abscopal effect after irradiation to the spine without surgery, chemotherapy, or immune checkpoint blockade therapy. Although there is a relationship between CCC and CA15-3 as reported in the past [13, 14], the fact that the primary tumor was not definitively diagnosed in the present case is a limitation.

After RT for the spine without systemic therapy, Ishiyama *et al.* reported an abscopal effect in a patient with renal cancer [1] and Leung *et al.* reported an abscopal effect in a patient with breast cancer [15]. The RT was combined with surgical treatment in those cases; however, we reported an abscopal effect without surgical treatment. The prescription doses in the cases reported by Ishiyama *et al.* and Leung *et al.* were 40 Gy in 5 fractions and 50 Gy in 25 fractions, respectively. In 2019, Azami *et al.* reported the abscopal effect after irradiation for breast cancer. They performed treatment for the breast cancer, pelvic bone metastasis, and lumbar spine metastasis without concomitant surgery [2]. In their study, the prescription dose to the lumbar spine was 39 Gy in 13 fractions, similar to the dose in our study.

There have been no reports of an abscopal effect in which only spinal metastasis was irradiated with radiotherapy alone, as in the present case. Radiation to the spine may have a tendency to be more successful than radiation to other metastatic sites. Hematopoietic stem cells in bone marrow throughout the spinal column may trigger innate immune recognition of the tumor through the release of cellular stress signals, even in the absence of tumor antigens. In fact, in a previous report on a review of ten articles by Mohamed *et al.*, it was hypothesized that irradiation of the spinal column may induce a greater abscopal effect than irradiation of other distant metastases [16]. They hypothesized that the mechanism of RT to ionize bone marrow, which naturally harbors lymphocyte populations, might be extrapolated to the abscopal effect of irradiation of spinal metastases. If irradiated hematopoietic stem cells in bone marrow could properly differentiate into lymphocytes that could travel throughout the body, it is possible that they could attack distant metastases. However, the hypothesis that radiotherapy of vertebral metastases causes immune activation is difficult to explain. For example, it is possible that hematopoietic stem cells are simply killed by palliative irradiation of the vertebral body. Further empirical and clinical validation is needed. In the present case, the patient was a heavy smoker, which may have affected bone marrow function. Normally, bone marrow hematopoiesis occurs in the spinal column and pelvic bones, but in smoking patients, hematopoiesis may also occur in the extremities [17]. It may be necessary to consider how much of an effect these changes have had.

In the present case, as a result of pathological diagnosis from cervical lymph node metastasis, CCC was diagnosed, and all of the lesions were considered to be CCC of unknown primary, but no pathological autopsy was performed, and some doubt concerning the definite diagnosis remains.

## Conclusion

In this rare case, radiotherapy alone for an extracranial metastatic lesion of a vertebra resulted in an abscopal effect on both extracranial and intracranial lesions. It is notable that the abscopal effect in the intracranial lesions was weaker than that in the extracranial lesions.

## Data Availability

The data include individual patient data, but the data are available from the corresponding authors upon reasonable request.

## Abbreviations

BBB: Blood–brain barrier
CCC: Clear cell carcinoma
CT: Computed tomography
FDG: ^18^F-2-fluoro-2-deoxy-d-glucose
MRI: Magnetic resonance imaging
PET-CT: Positron emission tomography–computed tomography
RT: Radiation therapy
SUVmax: Maximum standardized uptake value
WBRT: Whole-brain radiotherapy
